# Testing for optic ataxia in a blind field

**DOI:** 10.3389/fnhum.2013.00399

**Published:** 2013-07-25

**Authors:** Aarlenne Z. Khan, Laure Pisella, Ludovic Delporte, Gilles Rode, Yves Rossetti

**Affiliations:** ^1^INSERM, U1028, CNRS, UMR5292, Lyon Neuroscience Research Center, ImpAct TeamBron, France; ^2^Mouvement et Handicap, Hospices Civils de Lyon, Inserm et Université de LyonBron, France

**Keywords:** hemianopia, Bálint's syndrome, reaching, updating, eye movements

## Abstract

Optic ataxia is a component of Balint's syndrome and is a disorder that results from damage to the posterior parietal cortex (PPC) leading to deficits in reaching and grasping objects presented in the visual field opposite to the damaged hemisphere. It is also often the case that Balint's syndrome is accompanied by visual field defects due to the proximity of parietal and occipital cortices and also due to the subcortical pathway relaying visual information from the retina to the visual cortex passing underneath the parietal cortex. The presence of primary visual defects such as hemianopia often prevents clinicians from diagnosing higher-level visual deficits such as optic ataxia; the patient cannot reach to targets he/she cannot see. Here, we show that through the use of a paradigm that takes advantage of remapping mechanisms, we were able to observe optic ataxia in the blind field. We measured reach endpoints of a patient presenting with left optic ataxia as well as a quadrantanopia in the left lower visual field in eye-static and eye-dynamic conditions. In static conditions, we first asked the patient to reach to targets viewed in her *non-optic ataxic* intact right visual field (fixating on the left of the target array). In this case, the patient showed undershoots equivalent to controls. Next, we asked her to reach to (the same) targets viewed in the upper left *optic ataxic but* intact visual field (fixating to the right of the target array). The undershooting pattern increased greatly, consistent with unilateral left optic ataxia. In dynamic conditions, we asked her to view targets in her good (right lower) visual field before reorienting her line of sight to the opposite side, causing the internal representation of the target to be updated into the opposite (ataxic) blind visual field. The patient then reached to the remembered (and updated) location of the target. We found errors typical of optic ataxia for reaches guided toward the quadrantanopic field. This confirmed that reaching errors depended on the updated internal representation of the target and not on where the target was viewed initially. In both the patient and the controls, the updating of target location was partial, with reaching errors observed subsequent to an eye movement made from left to right fixation positions being intermediate between the left and right static conditions. Thus, using this remapping paradigm, we were able to observe optic ataxia in the blind field. In conclusion, this remapping paradigm would allow clinicians to test for visuo-manual transformation deficits (optic ataxia) even when it is associated with hemianopia.

## Introduction

One of the unresolved issues for clinicians is how to distinguish between primary visual field deficits and those that are higher-level such as visual neglect or extinction as well as those pertaining to Bálint's syndrome (optic ataxia, simultagnosia, and gaze apraxia). Indeed, when a primary visual deficit in the contralesional field is associated with a visuo-motor transformation deficit (such as optic ataxia) or of visual attention (such as visual neglect or extinction) in the contralesional field, it is difficult to evaluate the contribution of low-level and higher-level visual deficits to performance in the contralesional visual field. This is particularly the case in the presence of hemianopia.

We took the opportunity to test a patient with unilateral optic ataxia as well as a visual scotoma in order to evaluate whether optic ataxia can be revealed in blind field using an eye-dynamic paradigm based on saccadic visual updating (Khan et al., 2005a,b). Through the use of this paradigm, we were able to bypass primary visual processing of external stimuli and thus to isolate the higher-level visuo-manual transformation processes, since the visual stimulus is presented in the ipsilesional (intact) visual field and the response is provided in the contralesional visual field.

Optic ataxia is a disorder that results from either unilateral or bilateral damage to the caudal part of the posterior parietal cortex (PPC), including the parieto-occipital and the intra-parietal sulci (Pisella et al., 2009) and is classically manifested as misreaching to objects using visual information (Perenin and Vighetto, 1988). A clinical diagnosis of OA involves testing for accurate reaching to targets presented in the peripheral left and right visual fields while the patient fixates at a central location (Vighetto and Perenin, 1981). With bilateral OA, patients misreach to targets in both visual fields with both hands, whereas with unilateral OA, patients generally misreach to targets presented in the contralesional field with their ipsilesional hand as well as to targets presented in both visual fields with the contralesional hand (Perenin and Vighetto, 1988), however there are cases where patients only have a field effect without any hand effect (Blangero et al., 2011).

Damage to the PPC results from a multitude of factors, including traumatic brain injury, cerebral aneurysms, hypoxia, poisoning, neurological disease, and stroke damage, both ischemic and hemorrhagic. The PPC is particularly affected with injuries involving blood supply; it lies in a watershed region, an area that receives blood supply from the most distal branches of both the anterior cerebral and posterior cerebral arteries. As such, the occlusion of either artery can and does result in damage to the PPC. The posterior cerebral artery is also the primary blood supply to the visual cortex. Unilateral occlusion of this artery can lead to visual field defects such as homonymous hemianopia or quadrantanopia (Zhang et al., 2006). Therefore, the occlusion of the posterior cerebral artery can lead to both visual field defects as well as optic ataxia. Thus, when patients present with hemianopia, it is important to also test for unilateral OA within the same hemifield. However, it is impossible to test for reach errors associated with optic ataxia within the hemianopic visual field since patients cannot point to targets they cannot see, except in a blindsight paradigm (Perenin and Rossetti, 1996) in which pointing is not performed with enough accuracy to distinguish between the presence or absence of optic ataxia.

One solution is to take advantage of automatic updating mechanisms combined with memorized reach target locations. We previously showed that the reaching errors of unilateral optic ataxia patients (with no visual field defects) depended on where they were looking while reaching, rather than where they had viewed the target (Khan et al., 2005a). First, we established baseline errors while asking patients to reach to targets presented either in the impaired (left) or the intact (right) visual field, and found reach errors similar to healthy controls when they reached to targets presented in their intact visual field and much larger errors when reaching into their impaired visual field. Next, we asked patients to reach to a target presented in the impaired visual field, but first to make an eye movement to the opposite side before reaching to it, essentially updating the target location into the intact visual field. We found that the patients were able to reach to the target with minimal errors, just as if they had viewed the target in the intact visual field. Importantly for the current study, when they viewed the target in the intact visual field and then made an eye movement to the opposite side, they reached to the target as if they had viewed the target in the impaired visual field. In summary, we found that optic ataxia patients were able to accurately memorize the location of a target and to accurately update its location across eye movements. Previous imaging, behavioral, and patient studies have shown that these patterns of errors arise because targets for reaching are encoded and updated internally in a gaze-centered reference frame, i.e., relative to where the eyes are directed, both in the healthy (Henriques et al., 1998; Medendorp et al., 2003; Merriam et al., 2003) and damaged brain (Khan et al., 2005a,b; Dijkerman et al., 2006; Blangero et al., 2010). Therefore, we concluded that the specific deficit in OA was to convert this visual information (externally viewed or internally updated) into an accurate reaching movement (Khan et al., 2005a).

We propose therefore that using this updating paradigm makes it possible to diagnose OA within the hemianiopic visual field by testing for reach errors even when there are no visual targets present. To provide proof of concept, we show reaching errors in a patient presenting with quadrantanopia in the left lower visual field as well as unilateral left optic ataxia (as evidenced by reach errors to visual targets presented in the left upper visual field). Within the lower visual field, the pattern of reach errors changed when she reached to targets presented in the intact (right) visual field with or without an intervening eye movement. In summary, the patient showed reach errors consistent with OA to internally updated targets in the lower left visual field.

## Materials and methods

### Participants

**Figure 1:**
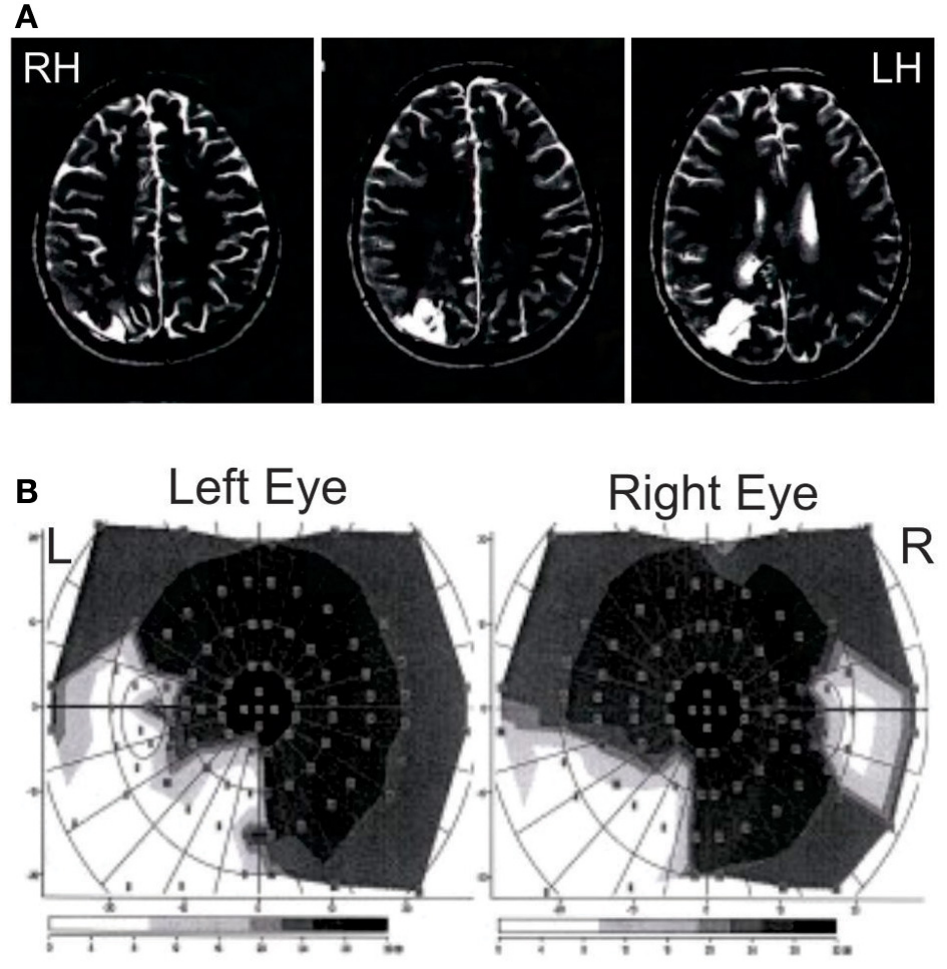
**Patient JG MRI and visual perimetry. (A)** MRI of patient JG. T2-weighted scans showing the damage after cyst removal (in white lower left) at the right parieto-occipital junction, including the caudal part of the intra-parietal sulcus and extending subcortically to the right lateral ventricle through the white matter. Scans are shown in radiological convention. **(B)** Goldman perimetry showing patient's quadrantanopia in the lower left visual field. She also showed somewhat large blind spots in both eyes.

Patient JR is a 23 years old right-handed female, who presented with ventricular meningioma that originated at the atrium of the lateral ventricle, removed in 2008, which was followed by the growth of a cyst, which was also removed. As a result of the surgery, she has a lesion that extends from the lateral ventricle into the white and gray matter of the right parieto-occipital junction including the caudal part of the intra-parietal sulcus, as can be seen in the MRI in Figure 1A. As a result of the damage to the white matter in the parietal area (Baum's loop), she has quadrantanopia in the lower left visual field (Figure 1B). She shows an impoverished visual memory (visual span of 4 items at Wechsler test) but preserved verbal memory (verbal span of 6 items at Wechsler test, score within the norm for the California Verbal Learning test: 14/16). She shows attentional deficits in disengaging from the right visual field using the Posner test (valid trials with target presented in the LVF: 359 ms, valid trials with target presented in the RVF: 373 ms, invalid trials with trials presented in the LVF: 438 ms, invalid trials with trials presented in the RVF: 369 ms) but no clinical neglect (no deficit in drawing from memory, copying the 5 items of the Gainotti test, line cancellation test).

She showed no executive dysfunction (Modified Card Sorting Test: 6/6), full preservation of language skills (object naming, verbal repetition, verbal fluency, search of contradictory words), and good execution of the Luria motor sequence, symbolic gestures and pantomimes, as well as no primary sensory and motor deficits at clinical assessment. Copying the Rey figure was difficult with 6 errors and a slow execution, a possible contribution of constructional apraxia (in addition to the primary visual deficit) to this difficulty seems confirmed by her own reports of problems with spatial orientation (she often get lost) and in dressing herself.

In addition, we also tested 7 neurologically intact participants (age range: 22–37, *M* = 28.14, all female, 6 right-handed). All participants provided informed consent to participate in the experiment which conformed to the Declaration of Helsinki for experiments on human subjects.

### Apparatus

**Figure 2:**
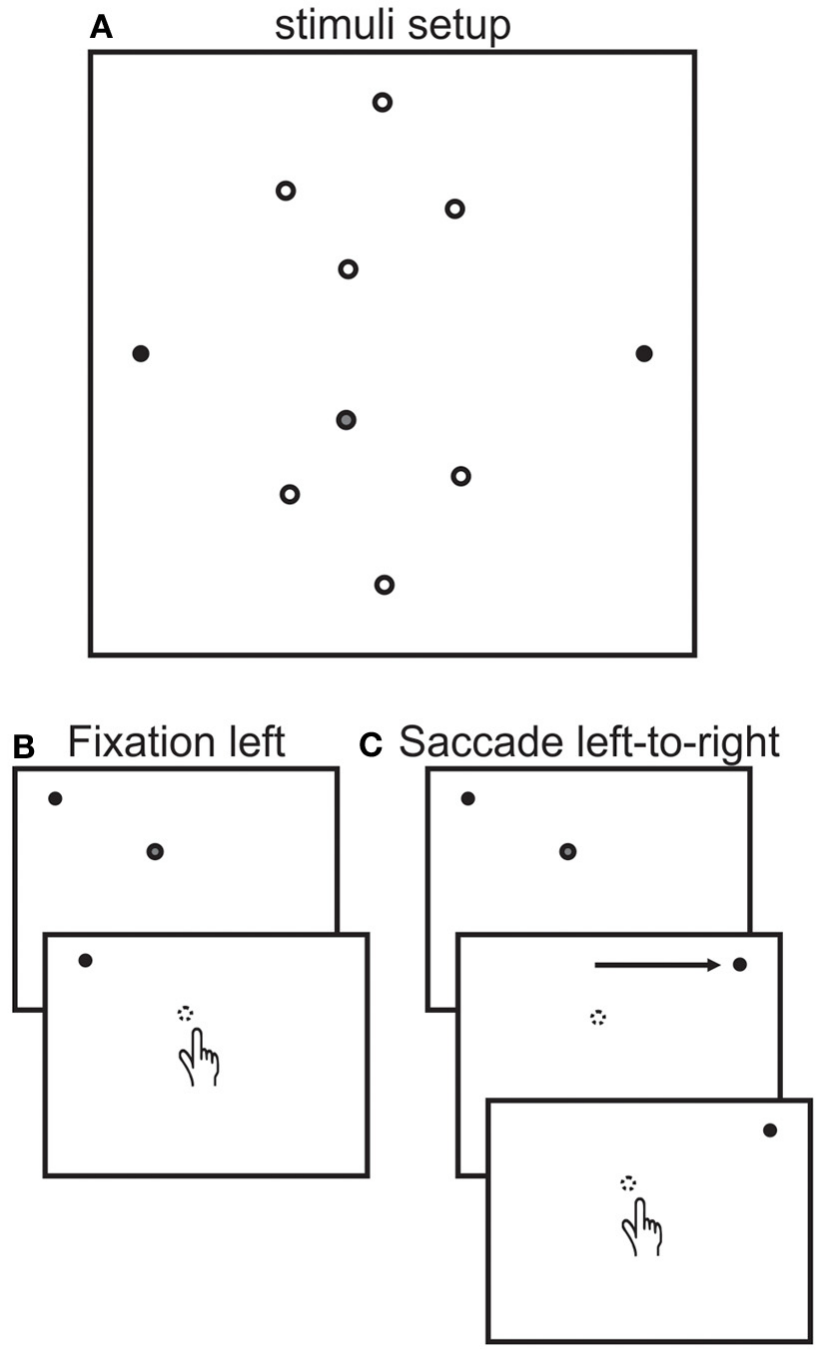
**Stimuli setup and trial sequence. (A)** Stimuli setup. Participants reached to one of 8 targets (open circles) while fixating on either the left (30°) or right (30°) fixation dots (black dots) or after making a saccade from the left to the right fixation dots. Targets were located at 15.5°, 9.5°, 5.5° left and 3.5° right of center and 7°, 13°, 15°, and 24° up or down of center. Target locations were illuminated with a laser pointer by the experimenter from behind the screen facing the participant. The gray filled target is the one used to describe task sequence next. **(B)** Fixation left sequence. Each trial began with the participant fixating on the left fixation dot. Next the experiment illuminated the target (gray filled dot). The participant then reached to the location of the target, which was automatically extinguished when the participant released the start trigger. The participant remained fixated on the fixation dot throughout the trial. The fixation right session was identical except that participants fixated on the right fixation dot. **(C)** Saccade left-to-right sequence. Each trial began in the same way as the fixation left sequence. Next the participant made a saccade from the left to the right fixation dot. For the patient and for targets in the lower visual field, the target was no longer visible once the patient made the saccade because of the patient's blind field. For the control subjects, the target was extinguished before the saccade was executed. After that, both groups reached for remembered location of the target.

Participants sat on a chair facing the center of a vertical blank white screen (Figure 2A) on which there were two fixation dots (filled black circles, located 30° left and right of center). The participant's eyes were located at a distance of 30 cm from the screen and were aligned vertically with the two fixation dots and centered between them horizontally. Participants reached to one of 8 different reach targets (open circles, 15.5°, 9.5°, 5.5° left and 3.5° right of center and 7°, 13°, 15°, and 24° up or down of center) using their right hand. Reach endpoints were measured using a video-based Vicon motion analysis system (Vicon Motion Systems) at 200 Hz, using a passive infrared reflector marker positioned on the participant's index finger. Participants began each reach movement by releasing a start trigger at the bottom of the screen, which extinguished the reach target (in all but one session for the controls, see below). Eye movements were monitored online using EOG for all but one control subject, who reported verbally whether she made any erroneous saccades (1 occurrence). Reaches were made in a lighted room and participants were able to see their hand during the entire experiment.

### Procedure

There were 3 different experimental sessions, (1) fixation left, (2) fixation right, and (3) saccade left to right. Figure 2B depicts the task sequence for a left fixation trial, showing only a portion of the screen with the left fixation and one target location (filled gray target in Figure 2A). The task sequence for the fixation left and right sessions were as follows. Participants were asked to fixate on the left (right) fixation (shown by the black dot) and place their hand on the start trigger. Thereafter, the experimenter illuminated one target (gray filled dot) using a laser pointer from behind the screen. The participant was unable to see the experimenter or the location of the reach targets except when illuminated by the laser pointer because of the bright lighting in front of the front of the screen. The participant was asked to reach to the target when it was illuminated while continuing to maintain fixation during the entire reach. The reach target was automatically extinguished as soon as the participant released the start trigger through the use of a custom-made switch connecting the laser pointer to the start trigger. There were no time limits for either the onset or the duration of the reach. Any trials during which the participant moved the eyes were repeated at the end of a block.

For the saccade left to right session (Figure 2C), participants were asked to make an eye movement from the left to the right fixation dot after the target was illuminated. The target was illuminated only after the participants were fixating on the left fixation dot. For the patient, due to her quadrantanopia, the target in the lower visual field would no longer be visible once she made the saccade, i.e., after the saccade the target would be in her blind field. However, for the controls the target would remain visible. Therefore, to make the task comparable, we modified the task for the controls as follows; each trial began with the illumination of the target for ~1 s while the participant was fixating on the left fixation dot. This time was approximately equivalent to the time that the patient took before making an eye movement after illumination of the target. The target was then extinguished, which signaled the control participants to make the saccade to the right before reaching. In this way, the target would also only be visible during left fixation and no longer visible during right fixation just as it was for the patient.

### Data analysis

The patient performed 7 trials for all conditions. For the left fixation condition, she reached to all 8 target locations. For the right fixation condition, she only reached to the upper 4 targets as the lower 4 were not visible due to her quadrantanopia. For the left-to-right saccade session, she only reached to the 4 lower targets, as these would be visible during left fixation and then disappear after the eye movement when she was fixating on the right fixation.

The controls performed 5 trials each for all 8 targets for all three sessions, fewer than the patient because we expected healthy neurologically intact participants to be less variable in their endpoint reaches, which was indeed the case (overall horizontal SD for controls = 2.21°−4.09°; patient = 8.33°).

Reach endpoints were determined as the point at which the finger touched the screen, i.e., the point at which movement velocity was 0, recorded as x, y, and z position in mm. In addition, at the end of the experiment each subject was asked to carefully align their fingertip to each target position (the laser pointer remained illuminated) and we used these positions as target positions for each subject. Using distance of the eyes from the screen (30 cm), these values were converted into degrees relative to screen center. Horizontal reach errors were calculated as the difference in visual degrees between the reach endpoint and the relevant target location. We measured horizontal reach errors because it is along the horizontal axis that we predict changes in endpoints, given that the fixation points varied from left to right. Based on previous studies showing an eye-centered representation of targets for reaching (e.g., Henriques et al., 1998), we expect that reach errors should vary as a function of the change in eye movements within the same dimension i.e., horizontal. Mean horizontal reach errors were calculated across all 8 target locations for controls and for the patient in the left fixation condition, and across all 4 target locations for the patient in the right fixation and the left-to-right saccade condition. JG's reach errors were compared against the control group using modified *t*-tests (Crawford and Garthwaite, 2002). In addition, we used the revised Standardized Difference Test (rSDT test; Crawford et al., 2010) to compare whether the difference in two scores for the patient was significantly different from the difference for controls. These tests were designed specifically to assess whether a single patient's performance falls within the range of controls, using the control group's mean and standard deviation. The tests provide a robust comparison of a single data point against a small group of controls for single case studies.

## Results

### Fixation sessions

**Figure 3:**
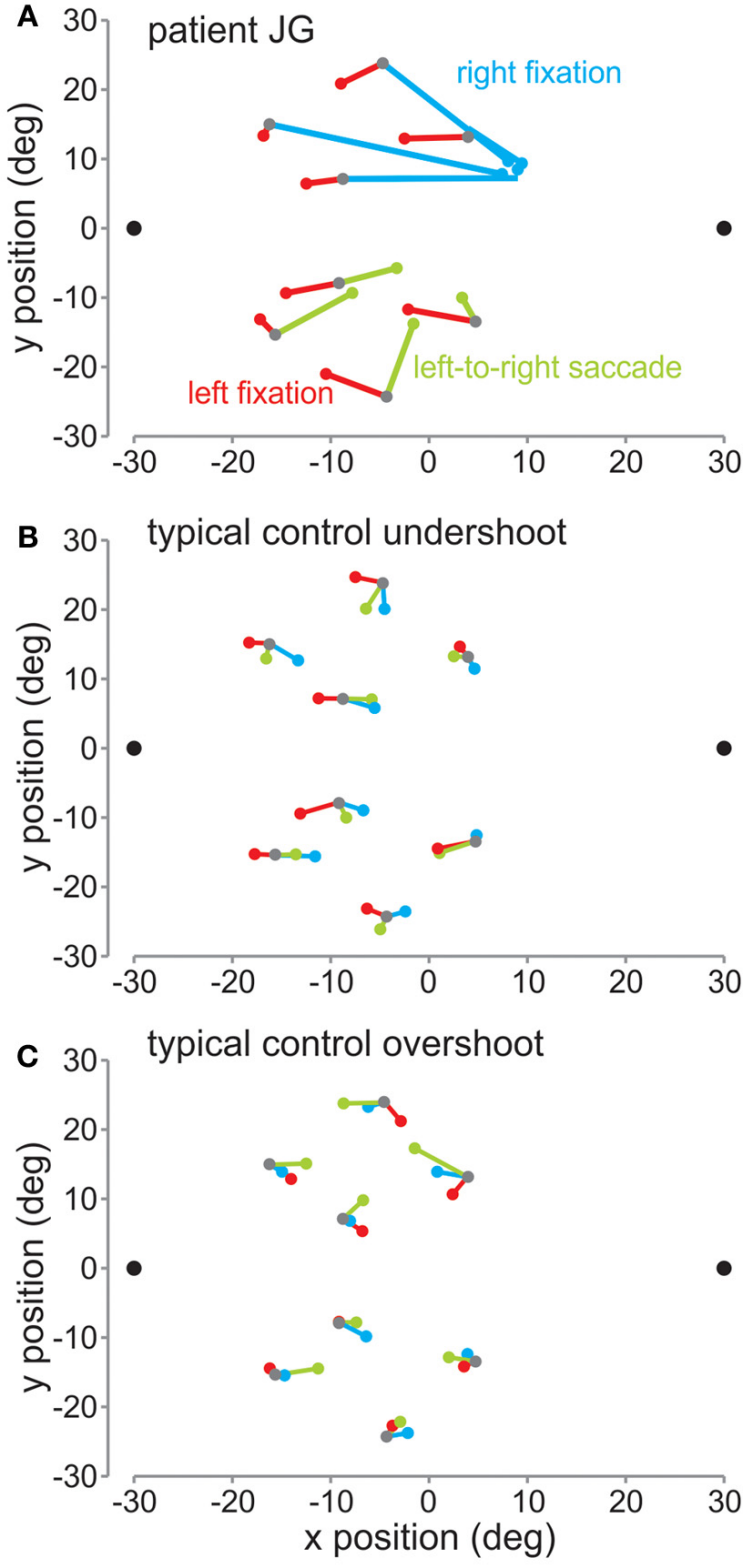
**Mean reach endpoints. (A)** Mean reach endpoints (in deg relative to screen center) for the patient's reaches for the left fixation (red), right fixation (blue), and saccade left-to-right (green) sessions. The 8 target locations are depicted by gray dots. The colored dots represent the mean endpoints for each of the conditions. They are joined by corresponding lines to their respective targets. **(B)** Mean reach endpoints for a typical control showing an undershoot pattern. **(C)** Mean reach endpoints for a typical control showing an overshoot pattern.

In Figure 3 are plotted the mean reach endpoints (in deg) for the patient (Figure 3A) and a control subject (Figure 3B) for the fixation left (red lines), fixation right (blue), and the saccade left to right (green) sessions across the different target locations. As can be seen, when the patient was fixating on the left fixation dot, the patient slightly undershot the target, as did this control. Within the control group, 5 of the seven control subjects followed a similar pattern, where they undershot the target, i.e., reached between the target and the fixation position. The other 2 control subjects tended to overshoot the target, i.e., reached further away than the target relative to the fixation position. A typical control with this pattern is shown in Figure 3C.

For left fixation, mean horizontal reach errors were not different for the patient from the controls [red lines: *t*_(1)_ = 1.96, *p* > 0.05], thus the patient did not show any OA in the right visual field. In contrast, when JG was fixating on the right fixation dot, she made very large errors reaching toward fixation, as has been shown previously for OA (Ratcliff and Davies-Jones, 1972; Buxbaum and Coslett, 1997; Carey et al., 1997; Blangero et al., 2010). Consistent with this, she showed significantly greater mean reach errors compared to controls [blue lines: *t*_(1)_ = 13.28, *p* < 0.01].

### Saccade left-to-right session

**Figure 4:**
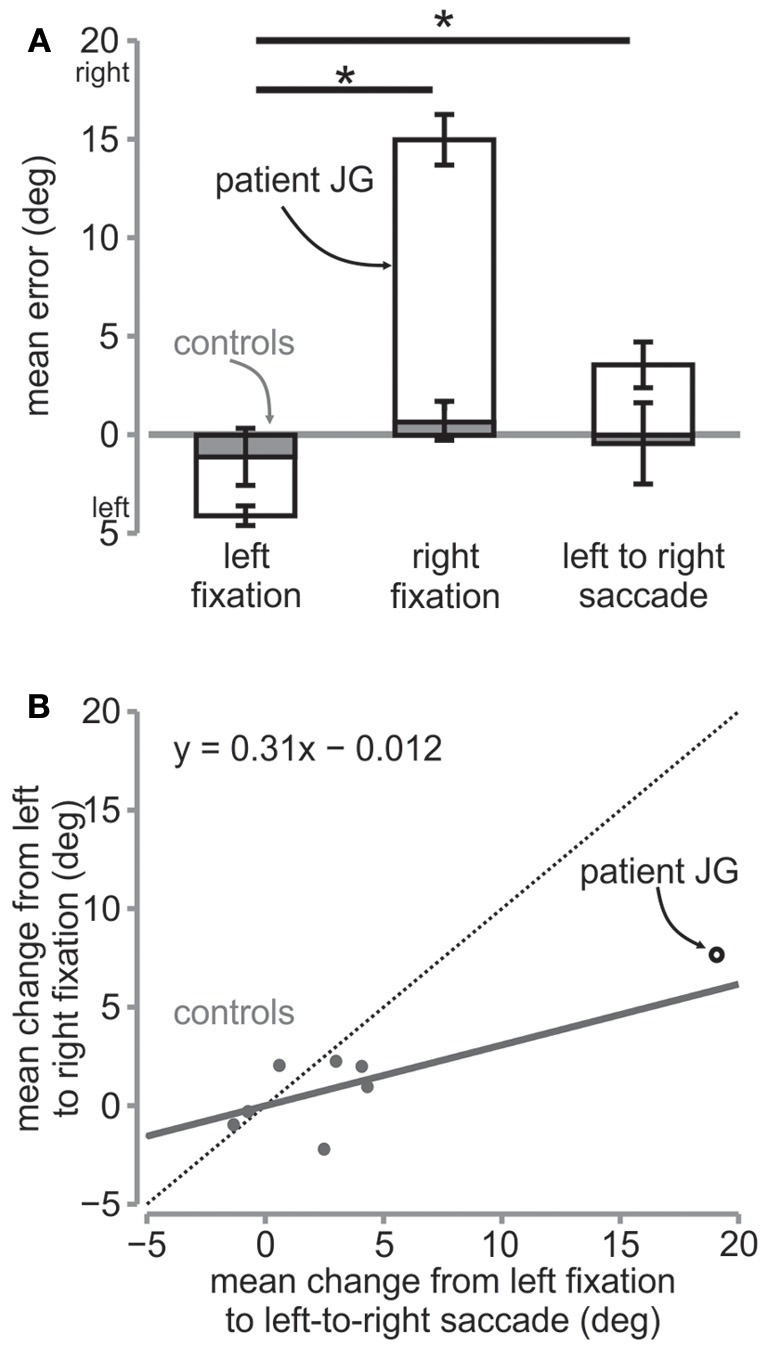
**Mean error and change in error. (A)** Mean horizontal error in degrees for the three sessions for the patient (open bars) and the controls (gray bars). The error bars for the patient are s.e.m. across trials, while those for the controls are s.e.m across controls. The ^*^ denotes significance at *p* < 0.05 across conditions for the patient. **(B)** Regression on mean change in errors. The mean change in error from left fixation to left-to-right saccade is plotted against mean change in error from left fixation to right fixation for controls (gray circles) and the patient (open black circle). The solid gray line is the line of best fit fitted to the control data. The regression equation is shown in the figure. The dotted diagonal line depicts the line of unity.

When the patient made a saccade from the left fixation to the right, reach endpoints shifted and were no longer similar to those during left fixation, even though the patient had viewed the targets in the right visual field. Rather the patient undershot the targets relative to the right fixation location, in the same way as during the right fixation session (Figure 4A). Reach errors were significantly different from the left fixation session for the patient JG [*t*_(58)_ = 6.34, *p* < 0.01] but not for the controls [repeated measures *t*-test, *t*_(6)_ = 0.82, *p* > 0.05].

For JG, we found that although the direction of the reach errors were qualitatively the same as during the right fixation, i.e., both undershooting relative to fixation, the reach errors were smaller than those during right fixation [left fixation = −4.12°, right fixation = 14.97°, left to right saccade = 3.54°; *t*_(54)_ = 6.6, *p* < 0.01, Figures 3A, 4A]. Similarly, controls' reach errors for the left-to-right saccade condition (−0.58°) were intermediate between the left (−1.12°) and right (0.66°) fixation, however they were not significantly different from right fixation [*t*_(6)_ = 1.47, *p* > 0.05]. We tested whether the difference in horizontal errors from left fixation to left-to-right saccade for the patient was similar to the controls and found a significantly greater difference for the patient than for the controls [patient = 7.65, controls = 0.54; rSDT test = *t*_(6)_ = 3.31, *p* < 0.01].

Since the patient showed greater errors overall, the greater difference can be expected and does not clarify whether the patient updated target locations the way the control subjects did. In other words, did the reach errors change in a similar way for the patient and the controls from left fixation when they made an eye movement from the left to the right fixation before reaching, i.e., intermediate between the two? To determine this, we plotted the change from left fixation to left-to-right saccade against the change from left fixation to right fixation for all subjects (Figure 4B). We then fitted a linear regression line to the control data (gray dots). The linear fit can be seen in the graph and represents the amount of updating taking place during the left-to-right saccade condition. If the location of the target was completely updated, the subject would point with the same amount of change as that from the left to the right fixation condition and so the data points should lie on the line of unity (dotted line), with a slope of 1. On the other hand, if there was no updating, the subjects would point the same as if they were fixating on the left, i.e., equal to no change from left fixation, and therefore there should be a slope of 0. Control subjects showed a slope of 0.31, suggesting that targets were partially updated across the eye movement. Importantly, as can be seen, the patient's data point also lies close to this slope, suggesting that she updated the targets in a similar way as the controls.

## Discussion

Here we took the opportunity to test a patient (JG) with unilateral optic ataxia associated with a visual scotoma in order to evaluate whether optic ataxia can be revealed in the blind field using an eye-dynamic paradigm based on saccadic visual updating (Khan et al., 2005a,b).

During the right fixation session, JG reached to the targets with a large error directed toward the fixation position. This is reminiscent of magnetic misreaching deficits previously shown, where patients have difficulties reaching away from the location of their current gaze. At extreme cases, patients reach toward their fixation location regardless of target position (Carey et al., 1997), however even for less extreme cases, there appears to be a consistent bias for errors in the direction of fixation (Buxbaum and Coslett, 1997; Blangero et al., 2010). It is interesting to note that there is a general trend to undershoot the target in the direction of gaze when patients (and controls) reach toward targets in lighted conditions, however in complete darkness, both groups tend to show the opposite pattern, i.e., they tend to overshoot the target away from fixation (Henriques et al., 1998; Khan et al., 2005a,b). It has been suggested that these differences might arise from whether or not participants have visual feedback of their hand before and during the reach (Dessing et al., 2012). For OA, visual feedback of their hand may bias their movements toward the fixation location to a greater degree than healthy participants.

For the left-to-right saccade session, JG did not reach with the same errors as she did during the right fixation session; although she consistently undershot the targets in the direction of fixation, she had smaller errors than in the right fixation session. Our previous study on unilateral OA patients also showed a similar but imperfect match between the magnitude of errors in the saccade and fixation conditions, where one patient reached with smaller errors and one with greater errors in the former compared to the latter condition (Khan et al., 2005a). Thus, the difference in errors could be because of imperfect updating mechanisms, due to her damage to more occipital areas, similar to bilateral OA who also showed delayed updating (Khan et al., 2005b). However, the magnitude of change in the saccade left-to right condition was similar between the controls and JG, suggesting that the underlying processes leading to the reach errors were similar. One explanation could be that because participants were reaching in lighted conditions, the presence of light may have provided some kind of allocentric reference frames against which to determine the target locations. In accordance, it could be that egocentric updating mechanisms play less of a role during lighted conditions.

Nevertheless, for patients with hemianopia both the target and allocentric cues might disappear during oculomotor exploration when they lie in the blind field. Our eyes move constantly to explore our visual environment. Objects therefore may lie in one visual field at one time and then in the opposite visual field during ongoing ocular exploration. In healthy subjects, because objects often remain static in the environment, they can use both current visual information (ego and allocentric) as well as internally updated information to locate the object (Vaziri et al., 2006). In contrast, in patients with hemianopia, ongoing ocular exploration will make objects disappear from conscious vision, therefore they have to rely more on updating mechanisms, which may result in more imprecise localizations. If they have intact visuo-motor and remapping processes they are nevertheless able to reasonably keep track of the presence and location of objects in order to interact with the environment. However, hemianopia combined with Bálint's syndrome may result in large consequences on daily activities. Indeed, they will have to deal with the additive effects of inaccurate action guidance toward visual targets as well as imprecision in localizing these targets or even obstacles with respect to the body and the environment. Therefore, it is important to diagnose Bálint's syndrome in the presence of hemianopia.

In addition, it is of considerable interest to understand what becomes of visual information viewed in the intact visual field when it is remapped into the blind field and how well it is used for higher-level transformations such as for reaching. Previous studies have suggested that updating is an important mechanism for hemianopia (Martin et al., 2007; Ritchie et al., 2012) and is possibly used to maintain visual stability for perception (Pisella and Mattingley, 2004) as well as to guide eye and hand movements. Here we show that targets are updated and used for reaching within the blind field in a very similar way as in the healthy brain.

In summary, the patient showed reach patterns consistent with optic ataxia in the quadranopic visual field by reaching to updated internal target representations. Based on these results, we suggest that this updating paradigm might be used to test patients with hemianopia for the presence of optic ataxia, a diagnosis that is otherwise not possible.

### Conflict of interest statement

The authors declare that the research was conducted in the absence of any commercial or financial relationships that could be construed as a potential conflict of interest.
